# Making individuals and needs visible: exploring the stigmatization against adolescents with mental health problems in photovoice practices

**DOI:** 10.3389/fpsyt.2026.1767130

**Published:** 2026-05-28

**Authors:** Qingyang Zhao, Yannan Peng

**Affiliations:** Social Work Department, The East Hospital Affiliated to Tongji University, Shanghai, China

**Keywords:** action research, adolescents, mental health, photovoice, stigmatization

## Abstract

**Introduction:**

Against the backdrop of mental health problems showing a trend towards an increasingly younger demographic, the stigmatization faced by adolescent patients has become increasingly prominent. Existing research predominantly adopts a microscopic and pathological perspective, often overlooking the agency of adolescent patients in expressing and resisting stigmatization.

**Methods:**

Utilizing a participatory action research approach, this study conducts an in-depth analysis of the stigmatization narratives of adolescent patients based on photovoice practices.

**Results and Discussion:**

The findings reveal that stigmatization associated with adolescent mental health problems is not a static label but is continuously constructed through multi-dimensional interactions between the individual and the self, family, peers, and society. It functions both as a pre-existing trigger contributing to the onset of mental health problems and as a post-diagnosis consequence arising from the illness, with the two interwoven in a cyclical relationship. Throughout this process, adolescent patients develop a set of struggle strategies ranging from individual self-help to collective mutual help. Moving forward, it is recommended to leverage the strengths of the photovoice to establish and reinforce an empowerment-oriented social support system, facilitating the stable recovery of adolescents with mental health problems.

## 1 Introduction

In recent years, mental health problems have emerged as a global public health challenge. The World Health Organization report “World mental health today: Latest data” reveals that over one billion people worldwide suffer from mental disorders, equivalent to one in every seven individuals experiencing varying degrees of psychological distress (1). These figures encompass 12 major disorders including depression, anxiety disorders, schizophrenia, and bipolar disorder, with depression and anxiety disorders being the most prevalent. Particularly noteworthy is the significant trend toward younger onset of mental health problems. The World Health Organization indicates that approximately one in seven individuals aged 10 to 19 worldwide suffers from mental disorders, accounting for 15% of the global disease burden in this age group (2). Depression, anxiety, and behavioral disorders are the primary causes of illness and disability among adolescents. According to the “Report on National Mental Health Development in China (2023–2024)” released by the Institute of Psychology, Chinese Academy of Sciences, young adults constitute a high-risk group for depression among adults. The detected depression risk rate in the 18–24 age group reaches as high as 24.1% (3).

Mental health problems not only impact individuals but also impose heavy burdens on families and society. Simultaneously, patients often endure dual pressures from society and themselves: stigma, discrimination, and the shame associated with illness. Goffman defines stigma as a profoundly demeaning attribute that transforms an individual from a whole, normal person into a tainted, degraded one (4). Link and Phelan further point out that stigma is a persistent problem that arises when labels, stereotypes, segregation, loss of status, and discrimination interact within power dynamics (5). While societal progress toward diversity can somewhat mitigate public rejection and aversion toward those with mental disorders, negative perceptions of mental illness rooted in historical and cultural contexts inevitably perpetuate the stigmatized status of individuals with mental disorders (6). Corrigan and Watson further distinguished between public stigma and self-stigma. Public stigma refers to the general public’s overall reaction to people with mental illness, whereas self-stigma is the process by which individuals internalize society’s negative beliefs about themselves, which may lead to a decline in self-esteem and self-efficacy (7). Before proceeding, it is necessary to clarify the terminology used in this paper. The terms “mental illness,” “mental disorder,” “mental health issues,” and “mental health problems” are often used interchangeably in the literature. However, in this study, “mental health problems” is adopted as an umbrella term to refer to the clinically diagnosed conditions experienced by adolescent participants (including, but not limited to, depression, anxiety, bipolar disorder, and obsessive-compulsive disorder). This choice reflects a person-centered and destigmatizing approach, furthermore, it better captures the variability and diversity of the psychological difficulties faced by adolescents. When citing policies, discussing classic works, or referencing others’ research, the terminology used by the original authors will be retained to ensure accuracy. In all other parts of this paper, the term “mental health problems” will be used consistently. Research have documented that people with mental illnesses face discrimination in various domains, including employment, housing, interpersonal relationships, and access to healthcare (8, 9). A cross-sectional study of 202 people with mental disorders found that as many as 87.6% of participants had experienced discrimination in at least one area of their lives over the course of a year (10). Mental health is an integral part of overall well-being. How to improve the physical and mental health of those with psychological disorders, reduce the stigma surrounding their conditions, and promote their social integration has thus become a critical issue.

In response to the increasingly severe mental health challenges among adolescents, the Chinese government has proactively implemented measures, with growing attention and investment directed toward mental health and psychiatric care. The “Healthy China 2030” Planning Outline and the National Health Plan for the 14th Five-Year Plan explicitly call for strengthening mental health service systems, intensifying public science communication on mental health, and enhancing training for relevant professionals. The promulgation and implementation of these policies have fostered a supportive environment for society-wide attention to individuals with mental health conditions, particularly among adolescents.

In China, existing research primarily focuses on the causes and treatment methods of adolescent mental health problems. Regarding etiology and influencing factors, personality traits (11), significant childhood events, parenting styles, and values may all contribute to the onset of adolescent mental health problems. Intervention methods typically encompass both pharmacological and non-pharmacological approaches, such as transcranial direct current stimulation (tDCS) (12), psychotherapy combined with medication, music therapy (13), exercise intervention therapy (14), traditional Chinese emotional care (15), and cognitive behavioral therapy (CBT). These interventions are primarily administered by physicians and psychotherapists. Regarding stigmatization and discrimination, researchers have focused more on stigmatization caused by or resulting from mental health problems, as well as how such stigmatization affects adolescents’ willingness and behavior to seek help. This reveals that traditional research predominantly adopts a pathological perspective, analyzing the causes of mental health problems and seeking solutions from micro-level perspectives such as individual patients and families. It pays less attention to meso- and macro-levels, overlooking adolescents’ agency in expressing their views and coping with difficulties, and failing to present the full picture of stigmatization and discrimination associated with mental health problems.

As an action research method, photovoice facilitates individual and societal change by organizing participants to photograph specific themes, engage in group discussions about the images, and share the stories behind them. Photovoice was pioneered by Wang and Burris with the aim of empowering marginalized groups, enabling them to document their life experiences and convey their perspectives to policymakers and the public (16, 17). Characterized by its emphasis on practice and participation, this approach enables marginalized groups to actively contribute, boosts participants’ motivation, and shifts researchers’ perspectives on issues (18). In recent years, photovoice has been increasingly applied in mental health research. Han and Oliffe provided a comprehensive review of photovoice studies in mental illness research, highlighting its potential to illuminate subjective experiences of recovery, suffering, and stigma (19). A systematic review and meta-analysis by Adeboye et al. demonstrated that photovoice interventions significantly improve depression symptoms, self-efficacy, and reduce social withdrawal among individuals with mental health conditions (20). In addition, photovoice has played a significant role in reducing negative stereotypes among healthcare providers, increasing their willingness to help, and promoting social engagement and empowerment among service users (21, 22).

Currently, in China, the photovoice has been applied in studies involving migrant mothers (23), community-based drug rehabilitation participants (24), individuals with disabilities (25), and rural mothers accompanying their children to school (26), but it has been less systematically employed to examine stigmatization or discrimination related to adolescent mental health problems. Adolescents are in a critical stage of identity formation, which makes them particularly vulnerable to the harmful effects of stigma (27). However, existing research has primarily focused on adult populations, resulting in a significant lack of understanding regarding how adolescents experience, internalize, and resist the stigma associated with mental health problems. Given adolescents’ unique developmental characteristics—including their reliance on peer relationships, ongoing identity formation, and gradually developing agency—there is an urgent need for participatory research methods that capture their voices and empower their involvement. Applying this method to the treatment of adolescent mental health problems provides young people with opportunities to express their inner voices. It facilitates the presentation of vivid, direct, and authentic narratives through the tool of “visual imagery,” promoting empowerment at individual, interpersonal, and societal levels to help adolescents achieve personal and broader societal change.

## 2 Methods

**Figure 1:**
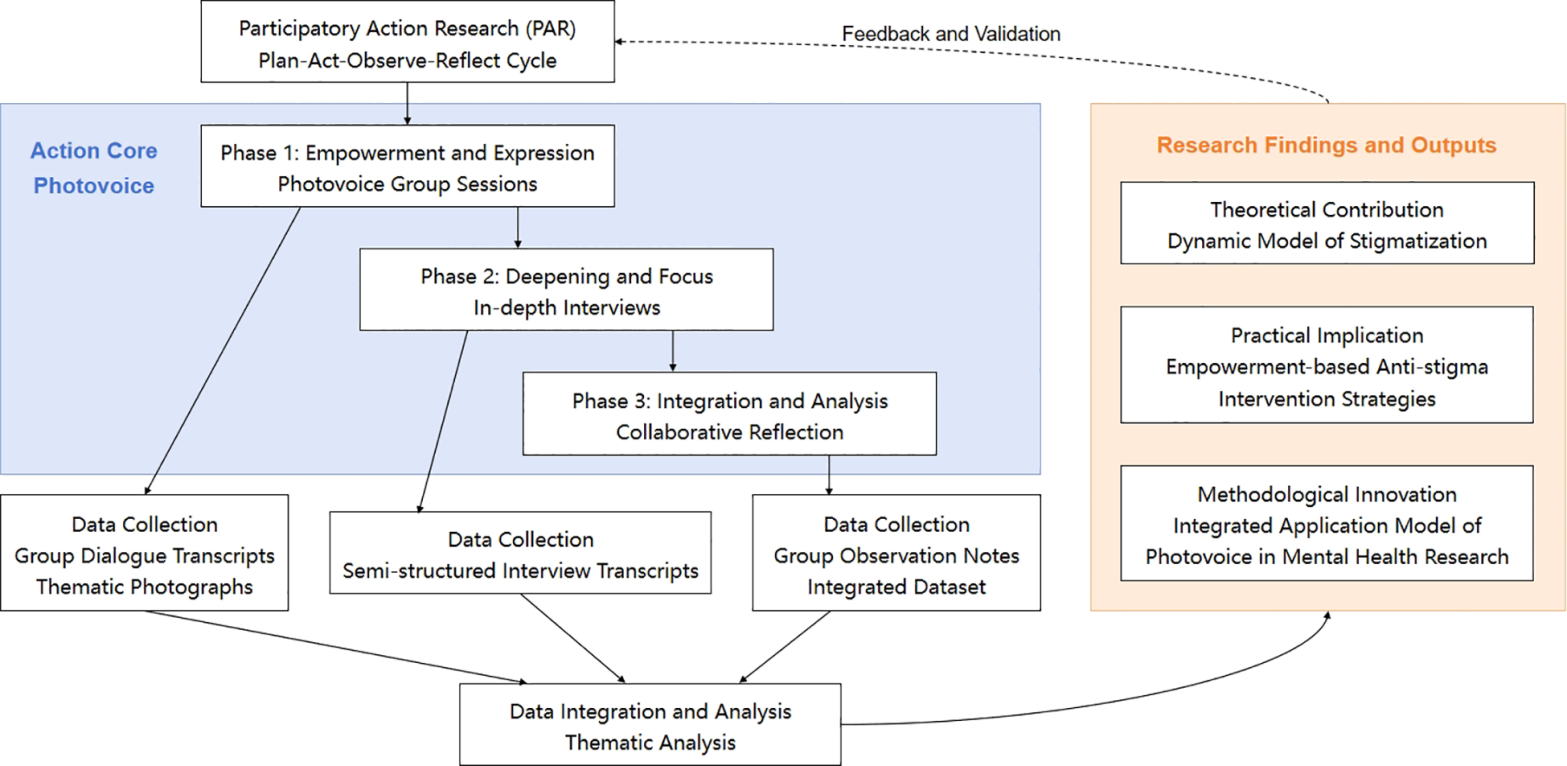
Research framework.

This study adopts a Participatory Action Research paradigm, aiming to deeply understand the stigmatization experiences of adolescent patients and stimulate their subjective expression through collaborative inquiry. The research employs the photovoice method as the core intervention and data collection technique, following the iterative “Plan-Act-Observe-Reflect” cycle to generate knowledge within dynamic group interactions. The research framework is shown in **Figure 1**.

### 2.1 Research setting and participants

The study was conducted from 2024 to 2025 in the Clinical Psychology Department of Hospital D. Adolescent inpatients were invited to participate in a continuous photovoice group series and were included in the action research. Inclusion criteria were (1): aged 10–19 years (2); diagnosed with a mental health problem (including but not limited to depression, anxiety disorders, bipolar disorder, obsessive-compulsive disorder) by a clinical psychologist (3); hospitalized in the Clinical Psychology Department (4); able to use a mobile phone to take or select photographs (5); willing to participate in group activities and provide informed consent. Exclusion criteria were (1): being in an acute episode of a severe mental health problem that prevented participation (2); having severe cognitive or communication impairments.

Ultimately forming an action research cohort comprising 32 adolescent patients and 8 family members. Among the adolescent participants, there were 12 males (37.5%) and 20 females (62.5%); age ranged from 10 to 19 years, with a mean age of 15.7 ± 2.0 years. Diagnostic distribution: depression 16 cases (50.0%), anxiety disorders 9 cases (28.1%), bipolar disorder 3 cases (9.4%), obsessive −compulsive disorder 3 cases (9.4%), and other 1 case (3.1%). Family member participants were parents or grandparents of the adolescent patients, including 5 females and 3 males. All participants provided informed consent; for minors, informed consent was also obtained from their legal guardians.

### 2.2 Action process and data collection

The research was integrated into a structured photovoice action process, consisting of three phases.

#### 2.2.1 Phase 1: empowerment and expression (photovoice group)

The researcher organized a continuous, rolling series of photovoice group sessions held weekly, with each cycle comprising four sessions. The group process adhered to the core steps of the photovoice method.

1. Thematic guidance and freestyle photography.
  Centered on themes such as “Myself in My Eyes,” “My Sources of Strength,” and “The Moment I Most Wish to Return To,” adolescent patients were encouraged to use their mobile phones to document life scenes related to their illness experiences and identity formation, or to select existing photos from their albums.
2. Group dialogue and critical reflection.
  Adolescents shared their photographed or selected images within the group. The researcher facilitated in-depth expression and collective discussion using the “SHOWED” facilitation guide: What do you see here? What is happening here? How does this relate to our lives? Why does this situation, concern, or strength exist? How can we become empowered through our new understanding? What can we do? Following each session, the researcher documented this process, generating dialogue transcripts.
3. Knowledge translation and advocacy.
  At the end of the group cycle, the researcher and adolescent patients collaboratively planned a photo exhibition. The photographs and accompanying narratives were curated into displays, serving as vehicles for social advocacy.

#### 2.2.2 Phase 2: deepening and focus (in-depth interviews)

Upon completion of the group sessions, purposive sampling was used to conduct one-on-one, semi-structured in-depth interviews with 16 adolescent patients and 2 family members selected from the total 40 participants. The sampling strategy considered diversity in gender, age, diagnosis type, level of participation in the group, and the richness of their sharing content. The interview guides were personalized based on core themes that emerged during the photovoice groups, aiming to delve into their life stories and explore the adolescents’ experiences of stigmatization. All interviews were transcribed verbatim.

#### 2.2.3 Phase 3: integration and analysis (collaborative reflection)

The researcher treated the dialogue transcripts from the photovoice groups, in-depth interview transcripts, group observation notes, and the participants’ photographs as an integrated dataset. Thematic Analysis was applied iteratively.

The coding process was as follows:

1. Open coding: The researcher read through all textual data and performed line-by-line coding, extracting initial concepts (e.g., “over-protection by family,” “peer avoidance”), resulting in 247 initial codes.
2. Axial coding: The research team discussed, compared, abstracted, and categorized the initial codes into themes. For example, codes such as “acting normal,” “deliberately hiding emotions,” and “proactive disclosure” were merged into “strategic concealment and exposure,” which was further classified under the category of “self-help at the individual level.” Ultimately, eight sub-themes were formed.
3. Selective coding: The sub-themes were further integrated to extract three core themes: “The Multi-dimensional Interactive Construction of Stigmatization,” “The Dual Role of Stigmatization,” and “Practical Strategies: From Individual Self-help to Collective Mutual Help”.

After coding, two participant representatives were invited to conduct member validation of the analysis results; their feedback was largely consistent with the thematic extraction, with only minor wording adjustments suggested. This analytical process embodies the “Observation and Reflection” stage of action research, ensuring the findings are grounded in the shared experiences of the adolescent patients.

### 2.3 Research ethics

This study was reviewed and approved by the Ethics Committee of The East Hospital Affiliated to Tongji University (Approval No. 2025YS-276). The trustworthiness of the research was ensured through ongoing peer debriefing, supervisory oversight, and researcher self-reflection. Ethical guidelines were strictly followed. Adolescent patients participated in the photovoice groups and shared photographs with informed consent. The researcher explicitly informed participants that they could withdraw at any time, and that their photographs would be used only for group sharing and authorized academic display. During group sessions, audio recording was not used. All dialogue and interview transcripts employed pseudonyms to protect participant privacy, and any identifiable personal information was removed.

## 3 The emergence of stigmatization: identity construction in multidimensional interactive relationships

Social interaction refers to the mutual influence and engagement between individuals or groups. Social interaction theory posits that human behavior is shaped by social interaction, not merely the result of internal factors (28). During social interaction, meaning is constructed through the use of symbols—particularly language—while the “self” and “social reality” are formed within this ongoing process of interaction (29). In this context, the stigmatization and discrimination surrounding adolescent mental health problems are not fixed, static labels. They are continuously constructed and reinforced through a series of dynamic social interactions, permeating the daily interactions between individuals and themselves, their families, peers, and society. Together, these interactions shape the patient’s “stigmatized identity”.

### 3.1 Self-interaction: internalized stigmatization and identity struggles

Interacting with the self is a process where cognitive and emotional experiences intertwine. Adolescents facing mental health challenges or suffering from psychiatric disorders are not merely passive recipients of stigmatization and discrimination. They often internalize external judgments through their self-interaction, simultaneously navigating a cyclical journey of self-identity exploration, doubt, and affirmation.

#### 3.1.1 The internalization of stigmatization

Numerous studies indicate that individuals with mental health problems internalize societal stigma, developing self-stigma manifested as self-blame, guilt, and self-deprecation. This study similarly found that when adolescents become aware of their mental problems, they may unconsciously transform society’s negative labels into self-denial, believing themselves to be “abnormal” or attributing their mental problems to personal character traits.

Right now, I’m just like this autumn leaf, slowly withering, hanging precariously on the tree, ready to fall at the slightest breeze. That breeze could be the mounting pressures from the outside world, but it could also be my own anxious, self-defeating, fragile, and pressure-sensitive nature. My resilience is poor. Many things that others see as trivial trifles leave me gasping for breath, feeling like the sky is falling. (A6).

Low self-esteem not only intensifies the inner suffering of adolescent patients but also hinders some from seeking professional help. At the same time, when a diagnosis is finally confirmed, the “illness” label may become internalized, acting as a catalyst for symptoms.

Since the first semester of my freshman year in high school, I’ve felt like I couldn’t keep up with my studies. I knew the material had gotten harder and my schedule was tighter than in middle school, but it seemed like that wasn’t the whole story. I couldn’t focus in class, and when doing homework, my mind felt completely blank—like it just stopped working. At first, I didn’t tell my parents, worried that if I went to the hospital and they actually found something wrong, what would I do then? (A7).

After receiving the diagnosis, I felt my condition deteriorate. My hands used to shake less severely than they do now. I don’t want others to know I’m sick, but the more I try to control it, the worse the shaking gets. I also experience night sweats, and I can’t manage many tasks properly. (A8).

Internalized stigmatization and discrimination act like invisible shackles, trapping adolescent patients in a cycle of self-denial.

#### 3.1.2 Identity conflict

Self-identity refers to an individual’s clear understanding and stable sense of self-identity, values, life goals, and other aspects. Adolescents are in a critical period for developing self-identity. They both want to fit in and yet refuse to blend into the crowd; they depend on peer support while also pursuing independence and self-reliance. Through integrating a series of internal and external interactions, they gradually construct a coherent and consistent self-perception and personality framework. “Who am I?” “Where do I come from?” “Where am I going?” are questions this group often ponders, yet they may not always find answers.

The person opening the door in the photograph is how others see me—tangible and defined. Yet the true self lies behind that door, a grayish-black shadow. Only by pushing through this door can I possibly find my true self. Sometimes I doubt whether this true self truly exists, for I don’t even know what form it might take. (A9).

Actually, I am an introvert, but I don’t want others to see me that way. I wish to appear as this tulip in their eyes—warm, cheerful, and optimistic. And I’ve truly made that happen, my classmates and friends all see me as a sunny, smiling person. (A2).

When adolescents experience mental distress, they may struggle with identity conflicts, wondering whether they are “sick or normal”, or choose to conceal their true selves, presenting a facade of normality to others. While this role-playing offers a sense of security, it also deepens their confusion.

I’ve been sick since high school, but I didn’t realize it at the time. For years, I’ve been living like a “joker” wearing a mask and trying to play a “normal person” in others’ eyes, completely losing myself in the process. Now I want to find myself again, but I don’t know where to do that. (A11).

### 3.2 Family-interaction: a contradictory space where care and stigmatization coexist

Interpersonal interactions within the family, involving parents, elders, siblings, and other relatives—are referred to as family interactions. These interactions form the cornerstone of adolescents’ social engagement, establishing the patterns through which they interact with individuals outside the family. For adolescents experiencing mental health problems, the family should serve as a core source of support. However, it can also become a significant source of stigmatization, revealing both complexity and contradiction.

#### 3.2.1 Support and over protection

When learning that their child has mental health problems, a significant number of parents interviewed expressed intense concern and self-reflection. They may conclude that they have set too high expectations, spent too little time with their child, or neglected their care. Consequently, parents and other family elders choose to adjust their parenting approach, offering adolescents more care and affection.

Before, whenever I told my dad I wanted to buy something, he’d just calmly analyze the pros and cons for me, or flat-out refuse. But recently, when I mentioned wanting to buy a few pencils, he actually asked me for the first time why I wanted them. I was so happy, it meant he was finally starting to pay attention to my feelings. (A1).

I specifically purchased several professional books on mental health, learning from them while putting the knowledge into practice as I accompanied him. Only then did I realize how poorly I had handled things before.(B1).

However, this concern and affection can sometimes go to the other extreme.

Now my family won’t let me do anything around the house, saying I’m sick and need proper rest. It makes me feel like a useless person. And they won’t let me hang out with my old friends either, afraid it might affect my mental state again. (A7).

Family members’ well-intentioned “over protection” inadvertently undermines the capabilities of adolescent patients, conveying an implicit message of stigmatization that suggests they are incapable of fulfilling normal social roles. This not only restricts adolescents’ social activities but may also foster feelings of inadequacy, intensifying their identification with the illness and reinforcing the label of “patient”.

#### 3.2.2 Pathological attribution and value denial

In some families, caregivers attribute all of a teenager’s emotions and behaviors to their “illness”, overlooking the genuine reasons and needs underlying their actions.

Last semester, I mentioned to my mom that I felt sleepy during class and couldn’t concentrate. She immediately assumed my anxiety must have flared up again. Hearing her say that made me anxious too. For the next few days, I kept telling myself I absolutely couldn’t zone out during lectures. But the more I focused on staying focused, the less I could absorb what the teacher was saying. And the less I could absorb, the more tense I became. (A6).

This pathological attribution often comes with a denial of the adolescent’s individual worth. Family members may unintentionally convey messages like “You can’t handle this because you’re sick”, which not only damages adolescents’ self-esteem and confidence but may also intensify their mental distress. Within this interaction pattern, young patients yearn for family understanding and support yet hesitate to express genuine feelings and needs for fear of being labeled “pathological”, creating a vicious cycle.

### 3.3 Peer-interaction: social distancing and labeling gaze

Beyond self-interaction and family-interaction, peer-interaction plays a crucial role in adolescents’ socialization process, profoundly influencing the formation of their identity and self-perception. Peer interaction refers to the daily exchanges and interactions among adolescents with their classmates, friends, and other peers. For adolescents experiencing mental health problems, peer groups can provide emotional support and understanding, yet they may also become a frequent source of stigmatizing experiences.

#### 3.3.1 From care to avoidance

This study found that peer group attitudes often undergo dynamic changes in phases: from initial curiosity and concern, to mid-stage confusion and uncertainty, eventually potentially evolving into stable distancing and avoidance. In the early stages, upon learning of a teenager’s mental health problems, peers may, out of care and goodwill, proactively inquire about the situation, listen to worries, offer comfort, accompany them through low moments, or share their own experiences to foster empathy. However, as time passes, some peers may feel overwhelmed due to a lack of professional knowledge and coping experience, or become concerned about their own emotional impact or being overly relied upon, leading them to gradually distance themselves. Some adolescent patients, meanwhile, may proactively withdraw from peer social circles due to internalized self-stigmatization.

At first, they tried to cheer me up, even making sure to include me in PE activities. But later, not knowing how to interact, they gradually stopped reaching out. (A9).

I know they care about me too, and I’m grateful for that. But sometimes it gets really awkward. I don’t want them to get talked about behind their backs just for hanging out with me, so I’ve decided not to hang out with them anymore. (A10).

Moreover, societal stigmatization of mental health problems subtly influences the attitudes of adolescents’ peer groups. Popular labels like “glass heart” “depressed netizens” and “Yu Yu Syndrome” may appear lighthearted and playful, but they can reinforce the “outsider” identity of young sufferers, leading to their exclusion from social circles.

My teacher criticized me for my poor test results, and I cried because I felt hurt. I thought the teacher didn’t recognize my worth. Then my classmates started saying I had psychological issues and was into “teacher-student romance”, claiming I cried because the teacher was married with kids and I couldn’t be his wife. (A2).

These speculations and labels prevent teenagers from showing their true selves to their peers, deepening alienation and division.

#### 3.3.2 Social rules: critique of “heterogeneity”

Just as in societal operations, interactions among adolescents within peer groups follow their own set of rules. When an individual’s personality, interests, or behavior deviates from the mainstream expectations of their peer group, they may experience subtle exclusion and pressure due to this “difference”, even without explicit hostile attacks, making it difficult to integrate.

I’m naturally outgoing and super enthusiastic with others, but I’ve come to realize that this straightforward personality has caused me a lot of hurt. My friends have told me several times that I “don’t know when to hold back”. I hope to learn how to gauge the right balance in my interactions with people. (A3).

The masked figure in the photo, wearing a hat, bears a resemblance to me, mysterious, with unconventional hobbies and a distinct aesthetic sense. Many times, others simply can’t grasp my expressions. (A5).

There are several cliques in my class who love gossiping about celebrities and stars. I’m not really interested in that stuff, and I’ve tried to fit in with them but just can’t seem to. (A8).

This “heterogeneity” is often perceived as deviating from the norm within peer groups, placing adolescent patients in a dilemma. On one hand, they yearn for acceptance and recognition, hoping to integrate into the collective, on the other hand, their uniqueness makes it difficult for them to fully conform to group expectations, thereby exacerbating the complexity of their mental health problems.

### 3.4 Social-interaction: mainstream culture and institutional exclusion

Social interaction refers to the process by which adolescents engage with their broader social environment. This interaction extends beyond the family and peer groups, encompassing multiple dimensions such as sociocultural factors and institutional norms, thereby forming the macro-level context for stigmatization.

#### 3.4.1 The standard for a “good child”

Within mainstream society, there exists a widely accepted evaluation system for “good child” behavior: excelling academically, pursuing wholesome hobbies, possessing a sunny disposition, demonstrating proper manners, actively participating in group activities with outstanding performance, and maintaining harmonious relationships … Among these, academic achievement stands as the most critical indicator. While this standard appears comprehensive and admirable, it cannot serve as a universal template for every individual. Adolescents are navigating a critical phase of self-discovery and worldview formation. As they explore through rough terrain and mature amid uncertainty, deviations from mainstream norms are inevitable. Those grappling with mental health problems are even more likely to “fall off track”, becoming “problem child” who fail to meet “good child” expectations, and consequently face exclusion and prejudice. Many participants in this study reported having experienced or continuing to endure the pressure stemming from these evaluation criteria.

I majored in finance, but to be honest, I lack the aptitude and intelligence for it. Studying has been incredibly difficult, and now (after getting sick) I feel completely lost in the material. My grades have dropped even further, and my advisor even called me into their office for a talk. The pressure is truly overwhelming. (A10).

At the same time, adolescent patients often internalize these standards, developing self-stigmatization. “Falling off track” leads them to constantly doubt themselves, repeatedly scrutinizing their behavior and actions—a process that further reinforces their self-stigmatization.

Actually, from elementary school all the way through seventh grade, my grades were consistently excellent. I could also play the piano, draw, and swim. Maybe it was because life was simpler back then, anyway, I excelled at everything and gradually set higher and higher standards for myself. Then, when I entered eighth grade, I realized I couldn’t keep up anymore. Whether it was academics or relationships, I felt overwhelmed and couldn’t figure out why. (A3).

I don’t hate knowledge; what I hate is this exam-oriented system. Lately, I’ve been seriously considering dropping out. Two voices are battling inside my head: on one hand, I genuinely can’t study anymore; on the other, I’m unsure if I can handle the consequences of leaving school. This internal conflict only makes me more anxious, leaving me feeling like such a failure. (A12).

A single evaluation standard overlooks the diversity and uniqueness of individual adolescents, and its impact extends to broader areas, hindering their rehabilitation process.

#### 3.4.2 Institutional exclusion in the environment

The educational system is the social institutional environment most frequently encountered by adolescent patients. It can provide the most timely support and assistance when mental health problems arise within this demographic, yet it may also inadvertently exacerbate stigmatization. Affected by mental health challenges, these adolescents often struggle to complete academic tasks. To secure sufficient time for treatment and emotional recovery, taking extended leave or withdrawing from school becomes their most common coping strategy. The interactions with teachers and peers both before leaving school and upon returning, along with their academic performance after resuming studies, significantly impact adolescents’ mental well-being.

Ever since my homeroom teacher suggested I take a leave of absence, he’s openly started to exclude me and make my life miserable. He keeps me out of all kinds of competitions and group activities, as if he’s trying to get rid of me as quickly as possible. (A6).

This year marks my second year on academic leave. I actually tried returning during my sophomore year of high school, but I only lasted three days before going home again. I realized that the longer I’m away from school, the harder it becomes to adjust back to the routine. I can’t follow the lessons, and my classmates spread rumors about me. I plan to take another year off before transferring schools. (A3).

When organizing activities, schools may restrict the participation of adolescent patients due to concerns about their physical condition or mental condition, which can make them feel marginalized. Furthermore, hospitals—an environment inextricably linked to the treatment and recovery process of adolescent patients, represent another significant setting where stigmatization and discrimination may arise. Many respondents reported that the stigma they experienced originated in hospitals.

During my last hospital stay, the management was extremely strict. Every hour, every minute, we had to follow their instructions on what to do. Activities were mandatory. My friend in the next ward couldn’t even use their phone. It felt like living in a prison. (A13).

They come to take my blood pressure at four or five in the morning, and turn off the lights early at night. Every day, I either stare at my phone or sleep, it feels so restrictive and stifling. (A12).

While this strict management model is implemented for therapeutic and rehabilitative purposes, it can easily lead adolescent patients to feel confined and monitored, further reinforcing their identity as a “special group”.

## 4 Dual role: both a result and a cause

A key finding of this study is that stigmatization play a dual role in adolescents’ mental health development trajectories: they emerge both after diagnosis, arising from interactions between adolescent patients and themselves, their families, peers, and society, as explained earlier, and before illness onset, serving as significant precursors and stressors for certain mental health problems. These two aspects often form a vicious cycle that is difficult to break free from.

### 4.1 Pre-existing stigmatization

Stigmatizing experiences in settings like schools and homes plague adolescents. Negative judgments, stereotypes, school bullying, family burdens, these pressures whether tangible or intangible, gradually accumulate, subtly eroding their mental well-being.

#### 4.1.1 School bullying and social isolation

School bullying is a common form of harm experienced by adolescents within educational settings, manifesting as verbal insults, exclusion, physical violence, or isolation and neglect. Some adolescents with mental health conditions had endured prolonged bullying prior to their diagnosis, or faced social isolation due to personality traits, sexual orientation, family background, or other factors. These persistent negative experiences directly contributed to the development of their mental health problems.

After returning to China to attend elementary school, I was frequently bullied by classmates. A group of students led the effort to isolate me, even forbidding students from other classes from speaking to me. Even our teachers adopted a passive-aggressive approach, ultimately letting the matter drop without resolution. This left me deeply hurt. (A1).

In third and fourth grade, their homeroom teacher was strict and disciplinarian, often subjecting her to corporal punishment. Some classmates would cheer her on from the sidelines and even mock her. Later in fifth grade, when they got a new homeroom teacher, the other students (who had been punished) were spared, but unexpectedly, she developed issues. We only learned later how deeply this incident had scarred her psychologically.(B2).

When adolescents experience school bullying, the response from schools, teachers, and parents is particularly crucial. If not handled appropriately, it can pose risks to the mental health of adolescents.

#### 4.1.2 A preview of family pressure

Before the onset of illness, the presence of strict parenting, emotional neglect, or high-conflict family dynamics creates a hidden, chronic, and subtle stigmatizing environment that significantly depletes adolescents’ mental resources. In some of the families interviewed, parents hold high expectations for their children. When these expectations go unmet, criticism and blame follow, causing adolescents to gradually lose confidence amid feelings of failure. In others, parents are preoccupied with work, failing to provide sufficient companionship and emotional connection. Still others are marked by tense atmospheres, constant arguments, and ongoing conflicts. These pressures accumulate over time, potentially triggering a range of mental health problems.

My uncle is a university professor, and my grandfather, aunt, and other family members have pinned all their attention and hopes on me, wanting me to become someone as accomplished as my uncle. Do you have any idea how much pressure that puts on me? I know I’m not good enough to meet their expectations, and this gap weighs heavily on me—I feel trapped both physically and mentally. Actually, I have a cousin too. I feel she’s the one with more potential, but my grandfather favors sons over daughters, so she can’t help lighten my load either. (A14).

My mother’s mood swings are unpredictable, while my father only knows how to pressure me relentlessly, forcing me to study without ever offering encouragement. Even when I ranked among the top students in my class, all I got in return was his criticism and dissatisfaction. My brother and I are simply in a transactional relationship where we do things for money. It’s safe to say I’ve never felt any kindness or warmth from my family. (A13).

These pre-existing stigmatization place adolescents in a prolonged state of repression and anxiety, gradually eroding their sense of self-identity while filling their minds with confusion and inner turmoil. This significantly heightens their risk of developing mental health problems. Moreover, such pre-existing stigmatization tend to be highly persistent. Even when adolescents have not yet exhibited obvious mental health problems, the negative effects they induce are difficult to eliminate in the short term.

### 4.2 The cycle of reinforcement between causes and results

This study argues that the stigma surrounding both the causes and results of illness rarely exists in isolation. Instead, they intertwine and reinforce each other in a relentless cycle within adolescents’ lives, forming a difficult-to-break closed loop.

On one hand, the mental pressure and negative emotions triggered by pre-existing stigmatization, such as the inferiority complex stemming from prolonged school bullying or the frustration arising from strict family education, serve as underlying factors that can precipitate mental health problems. When adolescents do develop mental health problems, the disease and its diagnostic label trap them in a new stigma trap—alienation from peers, societal prejudice, and other forms of stigmatization stemming from the illness. These new sources of pressure further exacerbate symptoms or trigger relapse, creating a vicious cycle that deepens the stigma’s impact.

On the other hand, adolescents’ internalization of stigmatization hinders their pursuit of professional help. They may delay seeking treatment, discontinue therapy, or conceal their condition, preventing effective disease management. This, in turn, reinforces societal prejudice and discrimination against this group.

## 5 Struggle: practical strategies from individual self-help to collective mutual help

Facing stigmatization across multiple dimensions, adolescent patients are not merely passive victims. They actively seek solutions, explore possibilities, and develop a range of creative individual and collective coping strategies to reclaim control over defining their own identities and steering their lives.

### 5.1 Self-help at the individual level: concealment, disguise, and active reconstruction

At the individual level, self-help represents the first step for adolescent patients in combating stigmatization. They attempt to avoid being labeled through various means, minimize potential harm, and rewrite their life stories.

#### 5.1.1 Strategic concealment and exposure

Depending on the audience, one may choose to conceal their true self and pretend to be “normal”, or shed the facade and reveal their authentic self, this constitutes the strategic tactics of concealment and exposure. To adapt to their environment and protect themselves, adolescent patients have evolved this survival tactic. Most report concealing their emotions and illness in unsafe settings like school, performing behaviors expected of “normal” people. Yet within trusted circles—family, close friends, or anonymous online communities, they shed this facade, freely expressing genuine feelings. For some, even the family home remains an unsafe environment requiring concealment.

Although I have many friends, I only share my illness with a few very close ones. In front of most people, I pretend to be perfectly normal. You never know how others might react or what they might do if they found out. Pretending to be normal is the only way I can feel safe. (A15).

Some adolescent patients choose to proactively disclose their condition, adopting a “strike first” approach to gain the upper hand. This strategy helps them avoid the potential harm of having to explain their situation passively, reduces the burden of explanation, and also offers the chance to connect with peers who share similar experiences.

I pinned the (illness) update to the top of my Moments so everyone could see it, sparing me from having to explain my leave of absence to each person individually. Every time I explained it, it felt like I was hurting myself. Now, surprisingly, no one asks anymore. After posting that update, a middle school classmate reached out to me—turns out she also has depression. We still chat regularly on WeChat. (A4).

Whether choosing to “conceal” or “expose” their condition, these are proactive decisions made by adolescent patients and crucial steps in their individual fight against the stigmatization of illness.

#### 5.1.2 Cognitive restructuring and meaning exploration

In the fight against illness, adolescent patients no longer blindly accept the labels imposed upon them by society. They begin to reexamine these disease-related tags, attempting to understand their experiences through different perspectives. They reconstruct their journey into a “rebirth journey”, which filled with pain yet also brimming with hope.

From last year until now, I’ve felt the world around me gradually sharpening into focus, slowly discerning the outlines of many things. It’s as if some external force is pulling me out of a black hole. Earlier this month, after lying in bed for over ten days, I finally mustered the courage to climb out of bed and step outside. There, I discovered my own capabilities: I could go out to admire the flowers, engage with others, and even uncover life’s small joys. I want to use a pair of scissors to cut off the mask on my face, to cut off the clown’s costume. Though this process is painful, I will tend to the wounds and embrace new beginnings. (A11).

After taking a leave of absence, my condition gradually improved. What used to be over two hours of repetitive hand-washing now takes less time, and my obsessive thoughts and behaviors have diminished somewhat. I’ve discovered that letting go of many pointless, stubborn thoughts is actually helpful. Lately, when compulsive behaviors resurface, I find ways to stop them, and sometimes I truly can. It’s like the river flowing beneath this bridge in the photo: though its course twists and turns, it will eventually pass through the darkness beneath the bridge and flow toward the rainbow in the sky. (A14).

At the same time, artistic pursuits such as writing and painting serve as their means to reconstruct their understanding and seek meaning. Through the creative process, they express their inner feelings, transforming the stigmatization of illness into creative energy, thereby achieving self-healing.

Back then, my classmates isolated and bullied me, so I withdrew from the outside world. Every day, I just made scrapbooks. These three scrapbooks are filled to the brim with my design sketches and book excerpts. Bedroom layouts, clothing, cars, multifunctional umbrellas, I even drew a relationship analysis chart of my classmates. I think it was precisely being isolated that sparked this endless stream of inspiration. Looking back at these scrapbooks now gives me a tremendous sense of accomplishment. (A1).

Positive meaning-making enables adolescent patients to gradually develop a more harmonious sense of self-identity, fostering self-acceptance and dismantling stigmatization.

### 5.2 Mutual help at the collective level: support and sharing

Beyond individual self-help, adolescent patients also develop strategies to combat stigma at the interpersonal and group levels. Online communities, inpatient peer groups, and trusted family and friends become vital sources of strength for them. Within these groups, illness ceases to be a solitary burden understood only by “me”, pain finally finds an audience for expression, and individual experiences coalesce into collective wisdom. Through this process, adolescent patients’ sense of agency and self-determination continually grows, enabling a transformation from “recipients of help” to “helpers”.

#### 5.2.1 Building a safe space

A sense of security forms the foundation of harmonious relationships. When members gather due to shared circumstances, engage in open sharing free from judgment, and experience deep understanding and empathy, a safe space is created. For adolescent patients undergoing hospitalization, peer-led support groups that involve regular participation among fellow patients serve as a vital safe space. Here, adolescents cease to be isolated, flawed individuals. Instead, through interaction, they form a community of action united by shared goals. Within the group’s dynamic, individual transformation occurs subtly yet profoundly.

Everyone listened attentively to others and responded thoughtfully. For the first time, I felt that bravely expressing praise for someone was an incredibly cool and wonderful thing, which I’d never dared to do before. It was everyone’s encouragement that gave me the courage. (A16).

We’ve been sharing a hospital room for nearly two weeks without much interaction, but today I realized how perfectly our personalities complement each other. Though we sometimes see things differently, we’ve learned to accommodate each other within the group. (A4).

Safe spaces also serve as platforms for sharing experiences and building collective knowledge. Adolescent patients and their families exchange rehabilitation experiences, resource information, and coping strategies for special circumstances within these groups. By offering diverse perspectives on challenges, they collaboratively construct a multidimensional framework for understanding.

What Mrs. Song just said really got me thinking. She’s probably around my mom’s age, and I’ve never discussed these things with my mom before. But today, hearing some of her feelings from a mother’s perspective, I think I’ll talk to my mom about it when I go home this time. Even though taking that step is hard, I’ll give it a try. (A5).

Beyond the safe spaces established within hospitals, online communities and close-knit circles of friends outside the hospital also serve as vital pillars for adolescent patients in their fight against illness and stigmatization. Many patients express that having friends who can hang out together, listen to their stories, and share frustrations makes them feel less alone and reaffirms their sense of worth. For adolescents with relatively harmonious family relationships, home can also serve as a safe space. The love and support from family members help them fight their illness with greater resolve.

#### 5.2.2 From “recipients of help” to “helpers”

The collective strength fostered within safe spaces not only alleviates adolescents’ feelings of isolation and helplessness but also ignites their courage and agency to confront adversity. This empowers them to transition from passive “recipients of help” to active “helpers”. This shift is first evident as they begin proactively sharing their experiences battling illness, offering guidance to new patients to better navigate the diverse challenges their condition may present.

I heard her say that this time, for some reason, it had flared up again. She felt there were no warning signs, it just suddenly got unbearable. So I shared my own experience with her. Actually, it’s been two years since my diagnosis, and I’ve had several relapses. When I’m in a good state, I feel completely alive again; when I’m not, I really can’t do anything. I told her never to stop taking her medication on her own, she must follow the doctor’s instructions. Relapses are normal with this disease, so she shouldn’t dwell on it. I shared a quote I found in a book: “What you accept disappears”. (A15).

My new roommate is only in eighth grade, and he’s also been bullied, his story is so similar to mine. I told him he can come to me anytime he wants to talk, because I’ve figured out how to deal with those annoying classmates now. I’ve become an expert through experience. (A5).

Some adolescent patients in recovery have stepped forward as “advocacy ambassadors”, participating in mental health awareness campaigns. They bravely share their personal stories with the public, drawing attention to individuals living with mental health problems and revealing the vibrant, warm lives hidden behind the stigmatization and prejudice surrounding these conditions.

After my last hospital discharge, I recall that it coincided with World Mental Health Day. Our school’s mental health club invited me to participate in a sharing activity—writing down my story and thoughts to post in the school’s mental health corner. At first, I declined because few people at school knew about my illness. But then I thought that if I don’t speak up, and neither do they, and no one does, we’ll never change others’ stereotypes about us. I refuse to let us forever feel like second-class citizens. (A16).

Adolescent patients have proven through their personal experiences that illness is not insurmountable. Even when trapped in the mire, they possess the power to transform their circumstances. Through mutual support within their community and social advocacy, they strive to live by the motto “My life, my choice”, while continuously working to foster a more inclusive and understanding society.

## 6 Conclusion and discussion

This study employed a participatory action research paradigm based on photovoice to explore the experiences of stigmatization among adolescents with mental health problems. Through a systematic analysis of photovoice groups, in-depth interviews, and photo narratives, the study found that the stigmatization experienced by adolescents with mental health problems is not a static label, but rather a dynamic process continuously constructed and reinforced through multifaceted interactions between the individual and their self, family, peers, and society. This finding resonates with and expands upon Goffman’s (4) concept of “spoiled identity” and Link and Phelan’s (5) definitional framework of stigmatization. For adolescents, the process of experiencing stigmatization is particularly pronounced due to their evolving sense of autonomy. Stigma manifests as a complex dual role: both a preceding factor contributing to adolescents’ mental health problems and a subsequent outcome arising after diagnosis. These two aspects intertwine, forming a vicious cycle of “stigma-illness-stigma”. Throughout this journey, adolescent patients develop a comprehensive set of coping strategies from individual self-help to collective mutual help, to protect themselves, reconstruct meaning in their lives, and distill personal experiences into collective wisdom. These strategies resonate with and complement the self-protection mechanisms proposed by Crocker and Major (30)—such as external attribution, in-group comparison, and selective devaluation—while carrying greater positive significance and contributing to the creation of a society free from prejudice and discrimination.

As a research tool in this study, the photovoice demonstrates unique value. The findings corroborated the quantitative evidence reported by researchers such as Russinova (31) and Flanagan (21), while further enriching these conclusions with qualitative data, indicating that the photovoice plays a significant role in reducing stigmatization, enhancing self-efficacy, and promoting social understanding. Through visual storytelling—a medium not entirely reliant on language, the agency of adolescent patients is activated, making them more willing to express the implicit experiences that profoundly impact their lives. Photographs serve as “catalysts”, facilitating deep sharing and collective reflection within group discussions, thereby transforming the research process itself into an empowering intervention.

At the same time, this study has certain limitations. The sample was drawn exclusively from a single hospital in one Chinese city, and lacked adolescents from diverse cultural backgrounds. The hospital setting itself is highly restrictive, which may influence adolescent patients’ experiences of stigmatization and their coping strategies. Furthermore, because participation in the photovoice group was voluntary, patients with severe conditions or marked social withdrawal were underrepresented. In the future, methods such as cross-cultural comparative studies and longitudinal research could be employed to investigate how the multidimensional interactions of stigmatization differ across cultural contexts and to examine the dynamic evolution of stigmatization. Furthermore, more diverse samples should be included to test the generalizability of the findings.

Breaking down societal stigmatization of mental health problems cannot be achieved overnight. It cannot rely solely on the proactive efforts of adolescent patients themselves but requires concerted action from multiple stakeholders. Leveraging the strengths of photovoice, photography exhibitions should be organized. Using photographic works created by adolescent patients as a medium, these exhibitions can present to the public the often-overlooked yet profoundly warm and powerful voices that exist within these communities. This approach helps deconstruct stigmatization and foster greater understanding. Furthermore, establishing an empowerment-oriented family and social support system is crucial. Serving as a “buffer zone” and “protective umbrella” for patients facing challenges, a robust support network can assist them in smoothly transitioning from patient to recovered individual.

## Data Availability

The original contributions presented in the study are included in the article/supplementary material. Further inquiries can be directed to the corresponding author.
